# Direct Observation of High Photoresponsivity in Pure Graphene Photodetectors

**DOI:** 10.1186/s11671-017-1827-0

**Published:** 2017-02-07

**Authors:** Yanping Liu, Qinglin Xia, Jun He, Zongwen Liu

**Affiliations:** 1 Hunan Key Laboratory for Super-microstructure and Ultrafast Process, School of Physics and Electronics, Central South University, 932 South Lushan Road, Changsha, Hunan 410083 People’s Republic of China; 2 Department of Materials Science and Engineering, University of California, Berkeley, California 94720 USA; 3 School of Chemical and Biomolecular Engineering, The University of Sydney, Sydney, NSW 2006 Australia

**Keywords:** Graphene, Photodetector, Photoresponsivity, Lightly p-doped substrate

## Abstract

Ultrafast and broad spectral bandwidth photodetectors are desirable attributable to their unique bandstructure﻿s. Photodetectors based on graphene have great potential due to graphene’s outstanding optical and electrical properties. However, the highest reported values of the photoresponsivity of pure graphene are less than 10 mA/W at room temperature, which significantly limits its potential applications. Here, we report a photoresponsivity of 32 A/W in pure monolayer graphene photodetectors, an improvement of over one order of magnitude for functional graphene nanostructures (<3 A/W). The high photocurrent generation in our devices can be attributed to the high sensitivity of graphene’s resistivity to a local change of the electric field induced by photo-excited carriers generated in the light-doping substrate. This dramatically increases the feasibility of using graphene for the next generation of photodetectors.

## Background

As a novel two-dimensional (2D) material, graphene possesses a unique conic band structure [1–3] and at low energy shows a linear energy dispersion of massless Dirac fermions [4–6] and thus is popularly considered for many optoelectronic and electronic applications [4]. For example, when the Fermi level of monolayer or bilayer graphene is tuned such that it is larger than half of the incident photon energy, graphene becomes completely transparent as a result of Pauli blocking [4–8]. This tunable property of graphene makes it highly suitable for many photonic devices [8–20]. A straightforward application of graphene, graphene photodetectors, has attracted numerous researchers, as graphene exhibits many advantageous behaviors compared to semiconductor counterparts [21, 22]. In addition to the aforementioned wide spectral absorption, with its high carrier mobility, graphene photodetectors are intrinsically capable of ultrafast operation not achievable by semiconductor-based detectors. The maximum bandwidth value of a reported graphene photodetector was 640 GHz [7], limited by the RC time constant of the measurement circuit. Additionally, the carrier extraction mechanism in graphene photodetectors is different from that of semiconductor photodetectors due to the built-in voltage at the metal–graphene junction [23]. Currently, photo-generated carriers extract from graphene photodetectors because the metal–graphene interfaces produce local potential variations [24]. However, the reported maximum responsivity in pure graphene photodetectors is very low (a few mA/W) [25], originating from the limited absorption (~2.3%) of the thin material, the small effective detection area of graphene sheet [4], and the short photo-generated carrier lifetime due to the gapless energy bands [4–8].

To date, work on creating graphene photodetectors with a high response speed and a broad spectral range at room temperature is still ongoing. More recent efforts concentrate on graphene functionalization techniques by creating a bandgap to enhance the photoresponsivity. However, not much work has been done on understanding and controlling the substrate effect on graphene photodetectors. Several known mechanisms contribute to photocurrent generation in biased graphene with a heavily doped silicon substrate [7, 24, 26]. Theoretical understanding and experimental investigations of generation mechanisms of photocurrents in graphene on lightly p-doped silicon substrates still have room for improvement, in how the thermoelectric and photovoltaic effects of graphene can affect the photocurrent. In this experiment, we investigate the mechanisms of photocurrent generation in biased graphene with a graphene field-effect transistor (FET) configuration fabricated on a lightly p-doped silicon/silicon oxide (Si/SiO_2_) substrate. We demonstrate a higher photoresponsivity at room temperature at visible range. The photoresponsivity value reaches a maximum of 32 AW^−1^ at room temperature measured from biased source-drain and back-gate voltage modulation, one order of magnitude higher than the values reported in recently published works. Our work potentially reveals the importance of the effects of the doping substrate for graphene photodetectors and offers insight on how to optimize pure graphene-based photodetectors and transistors for optoelectronic and electronic practical applications.

## Methods

Graphene photodetectors with an FET configuration were fabricated by mechanical exfoliation from bulk highly oriented pyrolytic graphite (grade ZYA, SPI Supplies). Photolithography and e-beam evaporation of 5 and 80 nm of chromium and gold, respectively, were used to create the source and drain electrodes. The sample of monolayer graphene was identified by optical contrast on the 300 nm SiO_2_ dielectric and confirmed with Raman spectroscopy. In order to detect the excessive photo field-effect, we used a lightly doped (p-type 10–20 ohm-cm) silicon dioxide on silicon substrate. In situ sample cleaning by the electric field were performed to clean the surface, enhance carrier mobility, and reduce intrinsic defects. After this treatment, the Dirac point of the devices were near 25 V. The photocurrent measurements were performed with a helium–neon laser with a wavelength of *λ* ≈ 632 nm at room temperature. Incident laser power values were fixed at 5 mW, and the frequency of the laser pulse could be adjusted from 5–5000 Hz. The laser spot diameter on the sample could be adjusted from *d* ≈ 1 μm to 1 mm using a microscope objective lens. The photoresponsivity data was collected with a semiconductor device analyzer (Agilent, B1500A). Considering the electric field enhancement attributable to the gate stack, we calculated that the absorption of the incident light in the graphene device was 2.5%. The photocurrent amplitudes shown are peak-to-background values throughout. The photoresponsivity measurements were carried out on multiple samples.

## Results and discussion

**Fig. 1:**
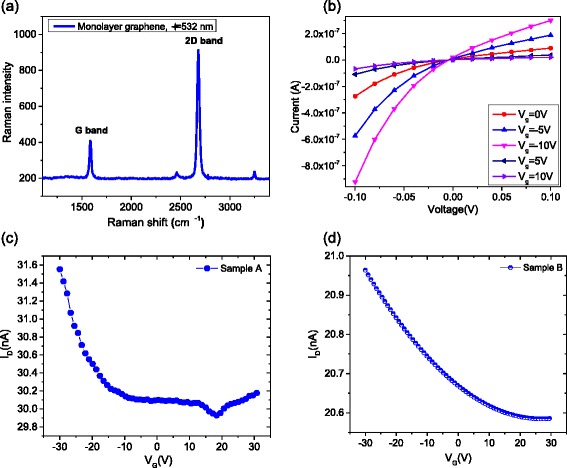
Electrical characteristics of our pure graphene photodetector. **a**. The characteristic Raman spectrum of the sample. **b** Back-gate voltage dependence of the current–voltage (*I*–*V*) characteristics of the graphene photodetector. **c** The source-drain current dependence of the back-gate voltage characteristics (transfer curve) of sample A. **d** The source-drain current versus the back-gate voltage bias of sample B

The FET devices were fabricated, and electrical characteristics of the graphene photodetectors were investigated. Figure 1a presents the current–voltage (*I*–*V*) characteristics of the graphene photodetector with back-gate bias (*V*_G_) varying from −10 to 10 V. Note that the current–voltage (*I*–*V*) with respect to *V*_s-d_ = 0 is asymmetric; this asymmetric behavior indicates that an intrinsic internal electric field (built-in potential) exists in the device. This plays an important role in the overall photocurrent response of graphene photodetectors, possibly as a result of both fabrication-induced defects and potential barriers between the edges of the graphene sheets. The inset in Fig. 1a shows the characteristic Raman spectrum of the sample with a clearly distinguishable G peak and 2D band. The ratio of the peak value *p*_2*D*_/*p*_*G*_ > 1 confirms that the sample is monolayer graphene. In addition, as the D peak is not seen in the Raman spectrum, the graphene sample must be relatively free of defects [27, 28]. Figure 1b shows the source-drain (*I*_s-d_) current dependence of the back-gate voltage (*V*_g_) characteristics (transfer curve) of sample A, and the inset shows the source-drain current (*I*_s-d_) versus back-gate voltage (*V*_g_) bias of sample B. The transfer curve indicates that the Dirac points (*V*_D_) of our devices were near 25 V.

**Fig. 2:**
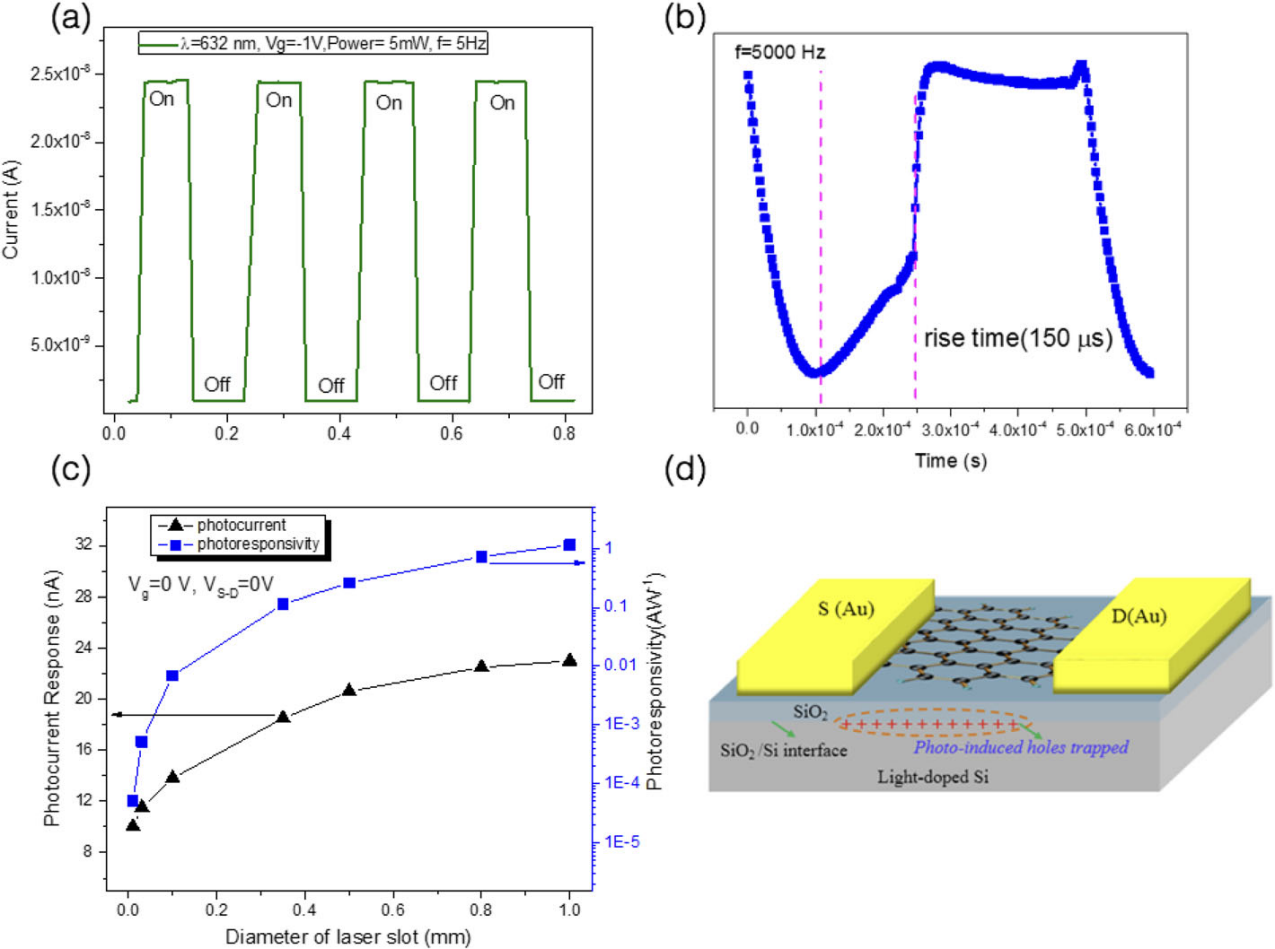
Optical characteristics of our pure graphene photodetector. **a** Time-dependent photocurrent measurements of sample A with a back-gate bias voltage of *V*_*G*_ = − 1*V* when optically pumped in the visible range (532 nm). The photodetector shows high photoresponses of approximately 1.15 AW^−1^ under the biased condition at room temperature. **b** The response time of the measured in one period of modulation with the laser illumination. **c** Photoresponse dependence on the laser spot size. **d** A schematic illustration of the positive charge accumulation at the interface of Si/SiO_2_ under light illumination without source-drain voltage bias, which effectively changes the back-gate voltage and induces a photocurrent

We investigated the photoresponsivity of the pure graphene photodetector device (refer to Sample A in the latter part of the paper) at source-drain voltage *V*_*s* − *d*_ = 5*mV* with biased back-gate voltage *V*_*G*_ = − 1*V*, with and without light illumination on the entire device at room temperature (see Fig. 2a). In these measurements, a focused laser beam (helium–neon gas laser, wavelength of 632 nm, laser spot size of 1 mm) is focused onto the device while the induced photocurrent is measured as a function of time. The device shows a high photoresponsivity of *S* = 1.15 *AW* ^− 1^(*G* = 97.98) at *V*_*s* − *d*_ = 5*mV*. Here, $S=\frac{I_{ph}}{\frac{S_G}{S_L}\cdot P}\left(A/W\right)$ and G is the gain of the photodetector defined as $G=\frac{I_{ph}/e}{\left(\frac{S_G}{S_L}\cdot P\cdot 2.3\%\right)/h\nu}\left(\nu =\frac{c}{\uplambda_{incident}}\right)$, where *I*_ph_ is the photocurrent [*A*], *P* is the incident laser power [*W*], *S*_G_ and *S*_L_ are the area of the sample and laser spot, respectively, and λ_incident_ is the laser wavelength. The laser-induced charge carrier generation and subsequent separation at the graphene–metal interface results in photocurrent generation. When light is incident on the interface between the graphene and the electrodes, the photo-excited electron–hole pairs are generated and accelerated in opposite directions by the internal electric field, thus generating an observable photocurrent *I*_ph_ = *V*_OC_/*R*_g_ (*V*_OC_ is open-circuit voltage produced across the carrier generation region, and *R*_g_ is the resistance of graphene).

To clarify the origin of the photocurrent, we investigated the photocurrent dependence on the laser spot size. This allowed us to clearly identify the photocurrent contributions from both the silicon substrate and the graphene–electrode interfaces, where internal electric fields are produced and separate the photo-generated carriers [26]. In real metal–graphene electrodes, the bending of the energy bands depends on the types and density of the surface states [29–32]. Figure 2b shows the photoresponsivity dependence on the laser spot size under the back-gate voltage bias of *V*_*G*_ = − 1*V*. As shown in Fig. 2b, the magnitude of the photocurrent *I*_ph_ and photoresponsivity are strongly dependent on the size of the laser spot. The photocurrent *I*_ph_ increases linearly with the laser spot size and saturates at spot size *d* = 1.0 mm. Such behavior indicates that the photocurrent originates from several different mechanisms [4, 25, 26]. We propose that the high photocurrent generation in our device arises from the high sensitivity of graphene’s resistivity to the local change of the electric field. In our experiments, lightly doped silicon substrates were used, resulting in the creation of a large vertical gate-like voltage on the substrate, increasing the total photocurrent. The local change of the electric field can be attibuted to the photo-excited carrier generation in the underlying lightly doped substrate. The inset in Fig. 2b represents the schematic illustration of the positive charge accumulation at the Si/SiO_2_ interface under illumination without a source-drain bias, leading to a photogating effect in the graphene FET [33, 34]. The graphene channel shows a high sensitivity to external electrostatic perturbation, as interfacial charge traps switch the gate voltage of the graphene FET, leading to efficient photocurrent. As the spot size decreases to a size matching the sample device, the photocurrent *I*_ph_ reduces to 10 *nA*(*S* = 0.8 *mAW* ^− 1^, *G* = 6.8 × 10^− 4^). This value agrees with the previously reported results of a low photoresponsivity of pure graphene of about 10 mAW^−1^ [25].

Owing to the work function mismatch between silica and silicon, the valence and conduction bands in silicon bend at the interface [26]. In our case of the p-type doped silicon substrate, the energy bands in silicon bend downward, leading to a triangular potential well for the electrons at the interface [35–40]. This downward bending of the energy bands near the metal electrodes enables the photon-generated electrons and holes to easily enter the metal without threshold energy barriers. This substrate effect would yield an enlarged vertical gate voltage, leading to high photoresponsivity [29]. Photo-generated electrons diffuse toward the interface, while holes are repelled away from the interface, allows for an additional negative voltage across the interface, creating a similarly biased negative gate voltage in the graphene field-effect transistor and changing the source-drain current. Interestingly, the spatial extension and the magnitude of the photo field-effect is determined by the substrate’s chemical doping [26]. For the heavily doped silicon, the carrier lifetime is relatively short. For intrinsically or lightly doped silicon (as in our case), the carrier lifetime is much longer, and the spatial extension of the effect can be as large as 1 mm. The intrinsic photocurrent decays fast when the laser spot is away from the graphene channel, but the photo field-effect can still be observed several millimeters away. Such properties enable us to estimate the magnitude of the photo field-effect via adjusting the size of the laser spot.

**Fig. 3:**
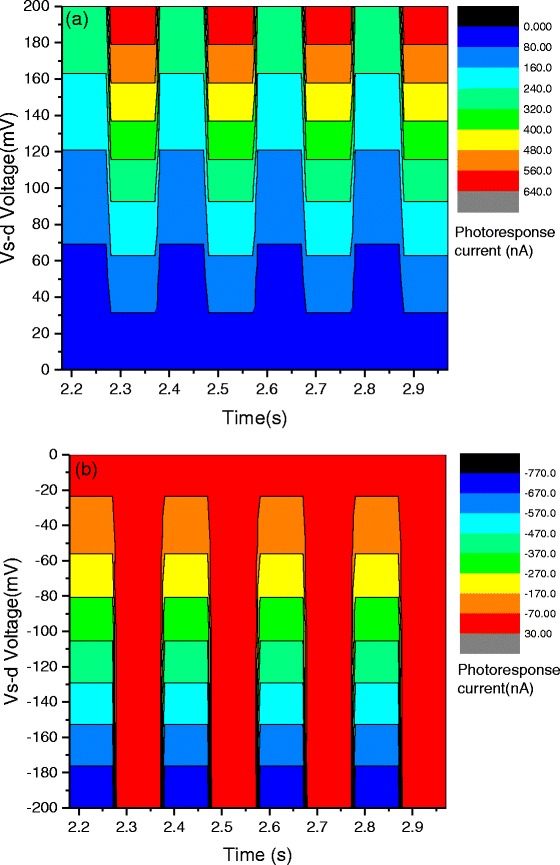
Photocurrent as a function of source-drain voltage. Time-dependent photocurrent measurements of our graphene photodetector with a positive (**a**) and negative (**b**) source-drain bias. These results indicate that the photocurrent can be tuned via a bias of source-drain voltage, indicating that a higher photocurrent can be readily obtained by applying a larger positive source-drain voltage

**Fig. 4:**
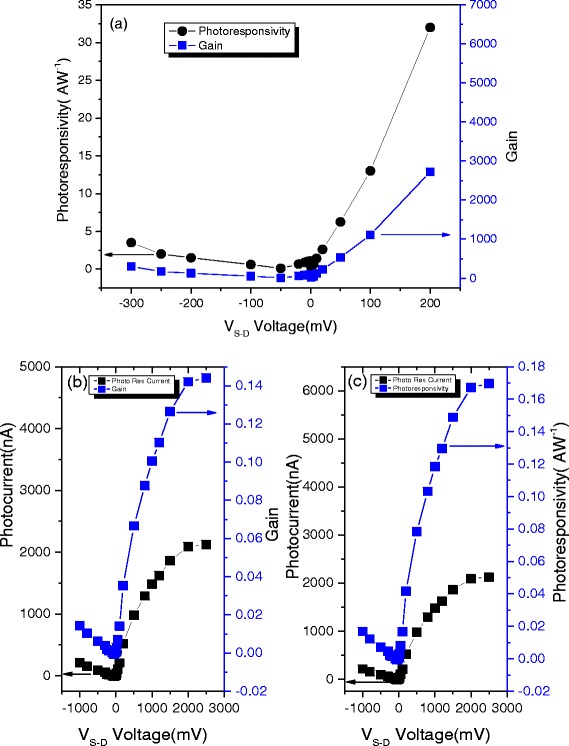
Photoresponsivity dependence on the biased source-drain voltage. **a** The gate voltage dependence of the photoresponsivity characteristics on the source-drain bias voltage. The decrease of the photocurrent with an increase of the gate voltage originates from the doping induced by the internal electric field. **b** and **c** present the source-drain voltage dependence of the external gain and photoresponsivity respectively without the substrate effect

The external photoresponsivity and gain can be further increased by applying a source-drain bias within the photocurrent generation path. The source-drain-dependent photoresponsivity characteristics (back-gate bias *V*_G_ stays fixed at 0 V) of sample A are shown in Fig. 3a biased positive and in Fig. 3b biased negative voltage. The photocurrents show a strong dependence on the biased source-drain voltage. An explanation for the increase of the photoresponsivity with the increasing source-drain voltage is that under light illumination, the overall current *I* is the sum of the photocurrent and dark current: *I* = *I*_*ph*_ + *I*_*d*_. Interestingly, the photocurrent *I*_ph_ increases with increasing source-drain bias voltage (applied to the two electrodes; one electrode grounded), but decreases for 0 > *V*_*s* − *d*_ > − 55*mV* and changes photocurrent sign at *V*_*s* − *d*_ < − 55*mV* (See Fig. 4). The critical voltage point *V*_*s* − *d*_ = − 55 *mV* demonstrates that the photocurrent becomes zero at this bias voltage regardless of the power of the laser illumination. This phenomenon indicates that the total electric field, which is the sum of the external electric field and the built-in field of the graphene channel, can be affected by applying a biased source-drain voltage.

Figure 4a presents the bias dependence of the external gain and photoresponsivity. As *V*_s-d_ increases, the gain and photoresponsivity increases linearly and the value is not being saturated. This observation potentially indicates that a higher photoresponsivity can be achieved with pure graphene devices. A maximum photoresponsivity of *S* = 32*AW* ^− 1^ was achieved at room temperature with a source-drain bias of *V*_s-d_ = 200 mV, representing an order of magnitude improvement over previously reported functional graphene nanostructure based photodetectors. Figure 4b, c presents the bias dependence of the external gain and photoresponsivity when the laser spot size is exactly the sample size, where the effect of the substrate can be screened. As *V*_s-d_ increases, photoresponsivity (*S*) and gain (*G*) rise linearly and saturate at *V*_*s* − *d*_ = 2*V*. A maximum photoresponsivity of *S* = 0.17 *AW* ^− 1^ is achieved at room temperature at a source-drain bias of *V*_s-d_ = 2000 mV, representing a 17-fold improvement over our previously reported value for our graphene photodetector at room temperature. The enhanced photoresponsivity with the addition of the substrate indicates that the lightly doped substrate significantly improves the functionality of the graphene photodetector. This result holds promise for prospective applications in a new era of high-performance optoelectronic devices.

**Fig. 5:**
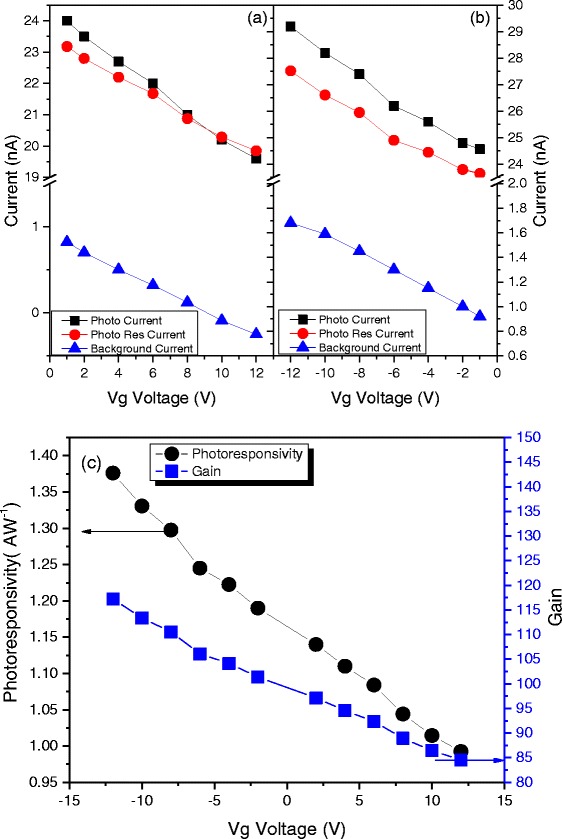
Photoresponsivity dependence on the back-gate voltage. The source-drain voltage dependence of the photocurrent of our graphene photodetector with a positive (**a**) and negative (**b**) back-gate bias voltage (−12 *V* ≤ *Vg* ≤ 12 *V*). These results indicate that the photocurrent can be tuned via the back-gate bias voltage. The increasing photocurrent with the decreasing back-gate voltage arises from the hole impact ionization effect with respect to the external electric field. As the gate bias decreases, more holes are involved in the impact ionization process to generate more electron–hole pairs. **c** Photoresponsivity and gain dependence versus back-gate bias voltage. A high photoresponsivity of 1.35 AW^−1^ is achieved at a *V*_G_ voltage of −12 V

Moreover, the external photoresponsivity and gain can also be enhanced by applying a back-gate bias within the photocurrent generation path and by augmenting the interaction between graphene and the incident light. Figure 5a, b displays photocurrent-generated bias dependence on the various voltage biases (−12 *V* ≤ *Vg* ≤ 12 *V*) applied to the silicon back-gate at *V*_*s* − *d*_ = 5*mV*. With a positively biased back-gate voltage, the photocurrent *I*_ph_ decreases with increasing back-gate bias voltage. In contrast, the photocurrent *I*_ph_ increases with the negative back-gate biases. Figure 5c presents the bias dependence of the external gain and photoresponsivity. A maximum external photoresponsivity of *S* = 1.38 *AW* ^− 1^ is obtained at a back-gate bias of *V*_*G*_ = − 12*V*. These observed values are larger than those reported in literature based on pure monolayer graphene-based photodetectors at room temperature with bias voltage. The increasing photocurrent with the decreasing back-gate voltage can be attributed to the hole impact ionization effect with respect to the external electrical field. As the gate bias decreases, more holes are involved in the impact ionization process, generating more electron–hole pairs. The observed results indicate that the back-gate voltage induced doping of the graphene channel and shifted the position of the Fermi level. Interestingly, the photo-induced current and the bias back-gate voltage are linearly related, indicating that higher photoresponsivity can be readily obtained by applying a larger negative bias back-gate voltage. Furthermore, the photoresponsivity and gain can be gate-modulated, providing a convenient on-off switching control for the graphene photodetector.

## Conclusions

In conclusion, we have demonstrated a highly effective method of increasing the sensitivity of a pure monolayer graphene photodetector by using lightly p-doped silicon dioxide on silicon as the substrate. Our pure graphene photodetector exhibits an increased photoresponsivity, one order of magnitude higher than that of similar graphene photodetectors in previous reports at room temperature. The observed phenomena in our experiments point to a clear pathway toward practical applications of pure graphene in the design of photodetectors that can be manipulated by back-gate (*V*_G_) and source-drain (*V*_s-d_) voltages. Moreover, the proposed configuration is superior to the previously reported structure, and a larger scale of the proposed device can be easily fabricated. Further device performance improvement can also be obtained through plasmonic nanostructures and waveguide-integrated configurations, providing that a method of growing high-quality graphene with high carrier mobility that is compatible with modern semiconductor technologies can be developed.

## References

[1] Nair RR (2008). Fine structure constant defines visual transparency of graphene. Science.

[2] Geim AK, Novoselov KS (2007). The rise of graphene. Nat Mater.

[3] Geim AK (2009). Graphene: status and prospects. Science.

[4] Koppens FHL (2014). Photodetectors based on graphene, other two-dimensional materials and hybrid systems. Nat Nanotechnol.

[5] Li ZQ (2008). Dirac charge dynamics in graphene by infrared spectroscopy. Nat Phys.

[6] He XY, Liu ZB, Wang DN (2012). Wavelength-tunable, passively mode-locked fiber laser based on graphene and chirped fiber Bragg grating. Opt Lett.

[7] Xia FN, Mueller T, Lin YM, Valdes-Garcia A, Avouris P (2009). Ultrafast graphene photodetector. Nat Nanotechnol.

[8] Fang ZY (2012). Graphene-antenna sandwich photodetector. Nano Lett.

[9] Pospischil A (2013). CMOS-compatible graphene photodetector covering all optical communication bands. Nat Photonics.

[10] Yoon D (2008). Strong polarization dependence of double-resonant Raman intensities in graphene. Nano Lett.

[11] Liu M (2011). A graphene-based broadband optical modulator. Nature.

[12] Zhou YX (2013). Tunable magnetoplasmons for efficient terahertz modulator and isolator by gated monolayer graphene. Phys Chem Chem Phys.

[13] Bao QL (2009). Atomic-layer graphene as a saturable absorber for ultrafast pulsed lasers. Adv Funct Mater.

[14] Sun ZP (2010). Graphene mode-locked ultrafast laser. ACS Nano.

[15] Xu JL, et al (2011) Performance of large-area few-layer graphene saturable absorber in femtosecond bulk laser. Appl Phys Lett 99:3672213

[16] Tan WD, et al (2010) Mode locking of ceramic Nd: yttrium aluminum garnet with graphene as a saturable absorber. Appl Phys Lett 96:3292018

[17] Rana F (2008). Graphene terahertz plasmon oscillators. Ieee T Nanotechnol.

[18] Bi L (2011). On-chip optical isolation in monolithically integrated non-reciprocal optical resonators. Nat Photonics.

[19] Xia FN (2001). An asymmetric twin-waveguide high-bandwidth photodiode using a lateral taper coupler. Ieee Photonic Tech L.

[20] Capasso F (2000). New frontiers in quantum cascade lasers and applications. Ieee J Sel Top Quant.

[21] Ishibashi T (2000). InP/InGaAs uni-traveling-carrier photodiodes. Ieice T Electron.

[22] Ishikawa Y, Wada K (2010). Near-infrared Ge photodiodes for Si photonics: operation frequency and an approach for the future. Ieee Photonics J.

[23] Meric I (2008). Current saturation in zero-bandgap, topgated graphene field-effect transistors. Nat Nanotechnol.

[24] Xia FN (2009). Photocurrent imaging and efficient photon detection in a graphene transistor. Nano Lett.

[25] Mueller T, Xia FNA, Avouris P (2010). Graphene photodetectors for high-speed optical communications. Nat Photonics.

[26] Freitag M, Low T, Xia FN, Avouris P (2013). Photoconductivity of biased graphene. Nat Photonics.

[27] Ni ZH (2010). On resonant scatterers as a factor limiting carrier mobility in graphene. Nano Lett.

[28] Ferrari AC, et al (2006) Raman spectrum of graphene and graphene layers. Phys Rev Lett 97:187401 10.1103/PhysRevLett.97.187401 17155573

[29] Loomis J, Panchapakesan B (2012) Large photocurrents in single layer graphene thin films: effects of diffusion and drift. Nanotechnology 23:265203 10.1088/0957-4484/23/26/265203 22699038

[30] Lu SX, Panchapakesan B (2006). Photoconductivity in single wall carbon nanotube sheets. Nanotechnology.

[31] Sze SM (1982) Citation classic—physics of semiconductor-devices. Cc/Eng Tech Appl Sci:28-28.

[32] Hatano T, Ishihara T, Tikhodeev SG, Gippius NA (2009) Transverse photovoltage induced by circularly polarized light. Phys Rev Lett 103:103906 10.1103/PhysRevLett.103.103906 19792315

[33] Ju L, et al (2014) Photoinduced doping in heterostructures of graphene and boron nitride. Nat Nano 60:348-352 10.1038/nnano.2014.60 24727687

[34] Liu CH, Chang YC, Norris TB, Zhong Z (2014) Graphene photodetectors with ultra-broadband and high responsivity at room temperature. Nat Nano 9:273-278 10.1038/nnano.2014.31 24633521

[35] Nicollian, Eh and Brews, Jr. MOS(Metal Oxide Semiconductor) Physics and Technology (Wiley, New York, 1982)

[36] Gabor NM (2011). Hot carrier-assisted intrinsic photoresponse in graphene. Science.

[37] Song JCW, Rudner MS, Marcus CM, Levitov LS (2011). Hot carrier transport and photocurrent response in graphene. Nano Lett.

[38] Ifuku R, Nagashio K, Nishimura T, Toriumi A (2013) The density of states of graphene underneath a metal electrode and its correlation with the contact resistivity. Appl Phys Lett 103:4815990

[39] Fang T, Konar A, Xing HL, Jena D (2007) Carrier statistics and quantum capacitance of graphene sheets and ribbons. Appl Phys Lett 91:2776887

[40] Nagashio K, Nishimura T, Toriumi A (2013). Estimation of residual carrier density near the Dirac point in graphene through quantum capacitance measurement. Appl Phys Lett 102:4804430

